# BMC *Pharmacology and Toxicology* reviewer acknowledgement 2014

**DOI:** 10.1186/s40360-015-0003-3

**Published:** 2015-05-12

**Authors:** Christopher Morrey

**Affiliations:** BioMed Central, Floor 6, 236 Gray’s Inn Road, London, WC1X 8HB, United Kingdom

## Abstract

The editors of *BMC Pharmacology and Toxicology* would like to thank all of our reviewers who have contributed to the journal in Volume 15 (2014).

**Peter Aduloju**

Nigeria

**Gustav Akk**

USA

**Catrin Albrecht**

Germany

**Adam Algren**

USA

**Hisham Aljadhey**

Saudi Arabia

**Maria Almeida**

Portugal

**Lamya Alnaim**

Saudi Arabia

**Nicolas Andre**

France

**Usman Bala**

Malaysia

**Alfred Balch**

USA

**Douglas Ball**

Philippines

**Desmond Bannon**

USA

**George Bayliss**

USA

**Vikhyat Bebarta**

USA

**Getenet Beyene**

Ethiopia

**Sagnik Bhattacharyya**

UK

**Marialuisa Bocchino**

Italy

**Jalel Boukadida**

Tunisia

**Peter Bruegger**

Switzerland

**Thomas Brunner**

Germany

**Miran Brvar**

Slovenia

**Martin Burtscher**

Austria

**Sarah Campbell**

USA

**Domenico Capone**

Italy

**Daniel Cardinali**

Argentina

**Michael Carvan**

USA

**Jose Castillo**-**Mancilla**

USA

**Luigi Cattel**

Italy

**Sarah Cavanaugh**

USA

**Thomas Chan**

Hong Kong

**Debabrata Chanda**

India

**Mario Chiong**

Chile

**Cristiana Cipriani**

Italy

**Francesco Clementi**

Italy

**Michael Coughtrie**

Canada

**Guillaume Darrasse**-**Jèze**

France

**Clara De Palma**

Italy

**Fabrizio De Ponti**

Italy

**Francesco Giuseppe De Rosa**

Italy

**Philip Debruyne**

Belgium

**Domenico Delfino**

Italy

**Shalinee Dhayal**

UK

**Robert Dickinson**

UK

**Eric Dietrich**

USA

**Kelly Dooley**

USA

**Vangelis Economou**

Greece

**Kamal El**-**Atrebi**

Egypt

**Ibrahim Elgendy**

Egypt

**Joseph Fadare**

Nigeria

**Rodrigo Franco**

USA

**Casper Franssen**

Netherlands

**Yubin Ge**

USA

**Alex Georgakilas**

Greece

**Kheirollah Gholami**

Iran

**Joel Goodman**

USA

**Andrew Lofts Gray**

South Africa

**Kristin Grimsrud**

USA

**Tyler Gums**

USA

**David Haas**

USA

**Katja Marja Hakkarainen**

Sweden

**David Hardman**

USA

**Thomas Hartung**

USA

**Yuya Hayashi**

Denmark

**Mai Helmy**

Egypt

**Robert Hendrickson**

USA

**Francisco Hernández**-**Luis**

Mexico

**Samuel Heyman**

Israel

**Lisa Hines**

USA

**Nguyen Quynh Hoa**

Vietnam

**Corinne Hohl**

Canada

**Hidehito Horinouchi**

Japan

**Lan Huang**

China

**Marilyn Huestis**

USA

**Shehnaz Ilyas**

India

**Shazia Jamshed**

Malaysia

**Piotr Janicki**

USA

**Zhenquan Jia**

USA

**Paul Jurasz**

Canada

**Keizo Kanasaki**

Japan

**Gopal Kannan**

India

**Barbara Kaplan**

USA

**Michalis Karamouzis**

Greece

**Marlies Karsch**-**Voelk**

Germany

**Jun**-**Ichi Kawabe**

Japan

**Ulrike Kemmerling**

Chile

**Tahir Khan**

Malaysia

**Alexander Koch**

Germany

**Shyam Kottilil**

USA

**Monica Lamberti**

Italy

**Cornelia Landersdorfer**

Australia

**Vincenzo Lariccia**

Italy

**Lawrence Lash**

USA

**Ping**-**Ing Lee**

Taiwan

**Zhoumeng Lin**

USA

**Kenneth Litwak**

USA

**Juan Carlos Lopez**-**Delgado**

Spain

**Rodrigo López**-**Muñoz**

Chile

**Henrik Lövborg**

Sweden

**Zuxun Lu**

China

**Yongjun Luo**

China

**Simona Magi**

Italy

**Tadeusz Malinski**

USA

**Wimolnut Manheng**

USA

**Alexandre Manirakiza**

Central African Republic

**Chiedza Maponga**

USA

**David Martinez**

Spain

**Ulrike Mende**

USA

**Christine Metz**

USA

**Martin C Michel**

Netherlands

**Kathryn Momary**

USA

**Ichiro Morioka**

Japan

**Danina Muntean**

Romania

**Chris Murgatroyd**

Germany

**Celso Vataru Nakamura**

Brazil

**Eiji Nemoto**

Japan

**Detlef Neumann**

Germany

**Seiji Niho**

Japan

**Stefania Nobili**

Italy

**Alfredo Nunziata**

Italy

**Fernando Palhano**

Brazil

**Giorgio Palu**

Italy

**Vasileios Papavasileiou**

Greece

**Kevin Park**

UK

**Davide Pastorelli**

Italy

**Martin Payne**

UK

**Angelo Peli**

Italy

**Napoleon Perez**-**Farinos**

Spain

**Andrei Pernambuco**

Brazil

**Jesus Pintor**

Spain

**Antonius Plagge**

UK

**Maria Cristina Da Silva Pranchevicius**

Brazil

**Matiram Pun**

Nepal

**Guoqing Qian**

USA

**Marek Radomski**

Ireland

**Shashi Ramaiah**

USA

**Emanuel Raschi**

Italy

**Marcia Ratner**

USA

**Cavan Reilly**

USA

**David Roberts**

UK

**Roberto Rodriguez**-**Roisin**

Spain

**Staffan Rosenborg**

Sweden

**Maurizio Rossini**

Italy

**Douglas Ruden**

USA

**Mitsue Saito**

Japan

**Yoshiro Saito**

Japan

**Laura Schnackenberg**

USA

**Angela Slitt**

USA

**Gabriele Stangl**

Germany

**Maciej Stepnik**

Poland

**Gabriele Stocco**

Italy

**Chris Stockmann**

USA

**Robert Matthew Strother**

New Zealand

**Ami Fazlin Syed Mohamed**

Malaysia

**Giovanni Tarantino**

Italy

**Pierre Teira**

Canada

**Maria Thurston**

USA

**Bennard Van Ravenzwaay**

Germany

**Andras Varro**

Hungary

**Raja Venkatasubramanian**

USA

**Charles Venuto**

USA

**Andrew Vernon**

USA

**Chris Vulpe**

USA

**Shigeo Wakabayashi**

Japan

**Niansong Wang**

China

**Jian Wang**

China

**Henrik Watz**

Germany

**Matthew Whittaker**

USA

**Bin Yang**

UK

**Yanqing Yi**

Canada

**Ebru Yildirim**

Turkey

**Tian Yu**

USA

**Anna Zaghini**

Italy

**Nathalie Zgheib**

Lebanon

**Naixia Zhang**

China

**Liming Zhou**

China

**Mario Zoratti**

Italy

